# Simultaneous Identification of Tc-99m-Sestamibi-Positive Autonomous Thyroid Adenoma and Adjacent F-18-Ethylcholine-Positive Parathyroid Adenoma in Patient with Graves’ Disease Using Real-Time Ultrasound Fusion Imaging

**DOI:** 10.3390/diagnostics15101262

**Published:** 2025-05-15

**Authors:** Theresa Leder, Philipp Seifert, Falk Gühne, Martin Freesmeyer

**Affiliations:** Clinic of Nuclear Medicine, Jena University Hospital, 07747 Jena, Germany; theresa.leder@med.uni-jena.de (T.L.); philipp.seifert@med.uni-jena.de (P.S.); falk.guehne@med.uni-jena.de (F.G.)

**Keywords:** ultrasound fusion imaging, parathyroid adenoma, sestamibi, ethylcholine

## Abstract

A 49-year-old female presented for nuclear medicine diagnostics of a sonographically suspected parathyroid adenoma dorsal to the cranial pole of the right thyroid lobe. The patient received Tc-99m-pertechnetate and Tc-99m-sestamibi (including SPECT/CT) scans, revealing no sestamibi uptake by the suspected parathyroid adenoma but a ventrally adjacent autonomous thyroid adenoma. Additional F-18-ethylcholine-PET/CT as well as subsequent Tc-99m-sestamibi-SPECT/US and F-18-ethylcholine-PET/US fusion imaging confirmed the suspected diagnosis of simultaneous autonomous thyroid adenoma and parathyroid adenoma. A blood analysis showed additional Graves’ disease.

**Figure 1:**
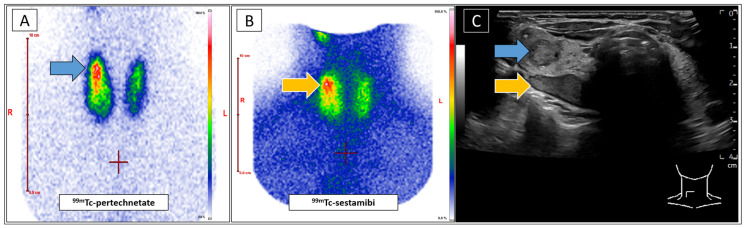
A 49-year-old female was referred to our clinic with a suspected parathyroid adenoma dorsal to the upper pole of the right thyroid lobe, as indicated by external ultrasound (US). A blood analysis revealed discrete hypercalcaemia, while the levels of parathyroid hormone and phosphate were within the normal ranges. No thyroid disorder was reported. An additional parathyroid scintigraphy should confirm the diagnosis before definite treatment, taking the mild biochemical changes into account. The patient underwent conventional standard-of-care diagnostics, including Tc-99m-pertechnetate (Figure 1A) and Tc-99m-sestamibi (Figure 1B) scintigraphies (Mediso Nucline TH-22, Münster, Germany) according to EANM guidelines [1]. Both scans showed increased uptake by the upper right thyroid lobe (Figure 1A blue arrow, Figure 1B orange arrow). Tc-99m-sestamibi uptake would be typical for a parathyroid adenoma, while focal Tc-99m-pertechnetate enhancement is unusual in such lesions [2]. A subsequent US (GE Healthcare LOGIQ E10, Milwaukee, WI, USA) confirmed the described nodule at the posterior thyroid margin (Figure 1C, orange arrow). Additionally, a second, as yet unidentified, intrathyroid nodule was identified in close proximity to the suspected parathyroid adenoma (Figure 1C, blue arrow). This configuration complicated the allocation of the metabolic information in correlation to the respective nodules. The focal Tc-99m-sestamibi enhancement likely indicated the suspected parathyroid adenoma (Figure 1B,C, orange arrows), while the intrathyroid nodule appeared to be associated with elevated Tc-99m-pertechnetate uptake, suggesting an autonomous thyroid adenoma (Figure 1A,C, blue arrows). Concurrently, the laboratory results revealed elevated TRAK values as well as moderately decreased TSH-levels. Accordingly, the presence of Graves’ disease was suggested notwithstanding the lack of concomitant clinical (no symptoms), sonographical (echonormal tissue without hyperperfusion), or metabolic (thyroid Tc-99m-pertechnetate uptake of 2.1%) indicators. (**A**): Planar Tc-99m-pertechnetate scan of the thyroid gland. (**B**): Planar Tc-99m-sestambi scan of the neck. (**C**): B-mode ultrasound of the thyroid gland (right lobe). The blue arrow indicates the suggested autonomous adenoma in the upper right lobe (**C**) and the corresponding focal Tc-99m-pertechnetate enhancement (**A**). The orange arrow indicates the suspected parathyroid adenoma (**C**) and its presumed corresponding sestamibi-enhancement (**B**). In view of these unexpected findings, Tc-99m-sestamibi-SPECT/CT (Siemens Symbia S, Erlangen, Germany) of the neck was performed, as it offers increased spatial resolution and diagnostic certainty (Figure 2A, SPECT MIP) [3]. Given the close proximity of the two identified nodules, additional Tc-99m-sestamibi-SPECT/US fusion imaging was indicated. This rare diagnostic approach has been demonstrated to facilitate precise metabolic differentiation between adjacent cervical lesions, achieved by augmenting metabolic 3D datasets with real-time US [4,5,6,7,8]. The scans revealed a clear correlation between the focally increased Tc-99m-sestamibi uptake and the autonomous thyroid adenoma (Figure 2B, grey arrow), which is a well-known clinical constellation [9]. However, the nodule at the back margin of the thyroid gland unexpectedly remained sestamibi-negative, indicating a coexisting Tc-99m-sestamibi-negative parathyroid adenoma. Studies suggest that low metabolic activity of the parathyroid adenoma, as in the present case, may be associated with reduced sestamibi uptake, thereby decreasing the sensitivity of the imaging modality [10,11,12].

**Figure 2:**
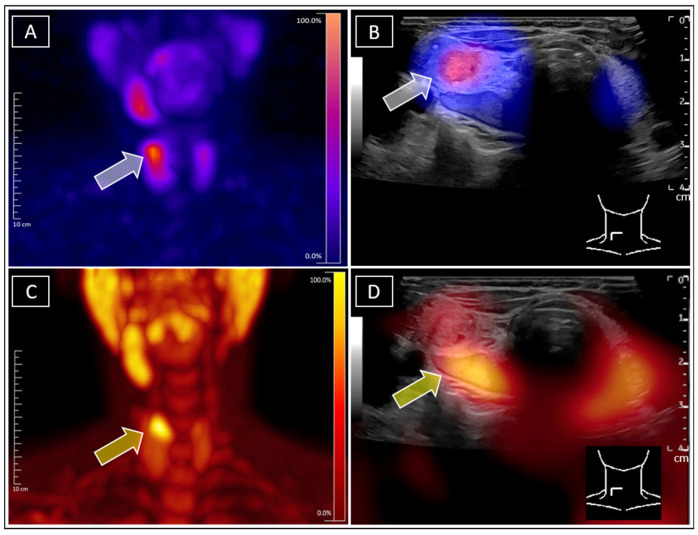
For further clarification, F-18-ethylcholine-PET/CT (Siemens Biograph Vision 600, Erlangen, Germany) was conducted according to local standards of care (86 MBq F-18-FEC i.v. (Life Radiopharma, Berlin Adlershof), 60 min p.i.), as it provides higher sensitivity in detecting parathyroid adenomas (Figure 2C, PET MIP) [13,14]. Due to the extremely rare constellation of the findings and the poor soft tissue contrast on CT, subsequent real-time F-18-ethylcholine-PET/US fusion imaging was performed in order to avoid further misconceptions. In analogy to SPECT/US, this approach is especially effective in cases with closely located nodes, enabling even more precise metabolic differentiation [15,16,17]. The images unambiguously revealed F-18-ethylcholine accumulation within the initially suspected dorsal parathyroid adenoma (Figure 2D, yellow arrow). Finally, the rare findings of concurrent Tc-99m-sestamibi-positive autonomous thyroid adenoma and adjacent Tc-99m-sestamibi-negative/F-18-ethylcholine-positive parathyroid adenoma were confirmed, unambiguously attributing the discrete hypercalcaemia to the parathyroid adenoma and therefore avoiding unnecessary further diagnostic steps with regard to potential differential diagnosis. This case illustrates a complex constellation with three concomitant diagnoses: Graves’ disease, autonomous thyroid adenoma, and parathyroid adenoma. The patient initially declined surgery and preferred drug treatment. Thyrostatic medication was started for Graves‘ disease. Although the TRAK levels decreased, the tendency towards hyperthyroidism remained. In agreement with the patient, simultaneous resection of the autonomous adenoma and the parathyroid adenoma are planned for the future. This case demonstrates the value of proper diagnosis for monitoring disease progression and ensuring appropriate therapy. (**A**): Tc-99m-sestamibi-SPECT of the neck, with the grey arrow indicating the focal sestamibi enhancement primarily attributed to the suspected parathyroid adenoma. (**B**): Tc-99m-sestamibi-SPECT/US unambigously allocating the focal sestamibi enhancement, indicated by the grey arrow, to the suspected autonomous adenoma of the thyroid gland. (**C**): F18-ethylcholine MIP of the neck, with the yellow arrow indicating the focal enhancement attributed to the parathyroid adenoma. (**D**): F-18-ethylcholine-PET/US, with the yellow arrow clearly allocating the ethylcholine-enhancement to the sonographically suspected parathyroid adenoma at the dorsal margin of the thyroid gland.

## Data Availability

Not applicable.
